# A deeper view of transmitted/founder viruses using 454 whole genome deep sequencing

**DOI:** 10.1186/1742-4690-9-S2-O58

**Published:** 2012-09-13

**Authors:** DC Tully, KA Power, H Bedard, CL Boutwell, P Charlebois, EM Ryan, NJ Lennon, M Altfeld, MR Henn, TM Allen

**Affiliations:** 1 Ragon Institute of MGH, MIT and Harvard, Boston, MA, USA; 2 Broad Institute of MIT & Harvard, Cambridge, MA, USA

## Background

Understanding the composition of transmitted/founder viruses that represent the primary target for vaccine-elicited responses is highly relevant to the design of an effective HIV-1 vaccine. The identification of transmitted and early founder viruses in primary infection has remained remarkably opaque, but recent advances in sequencing technologies have enabled a more comprehensive and sensitive assessment of the properties of transmitted/founder viruses.

## Methods

We applied full-length HIV-1 genome 454 deep sequencing of plasma virus to 22 clade B subjects from a cohort of men who have sex with men (MSM) identified during the earliest phase of acute HIV-1 infection (Fiebig stage II to III). Using novel assembly and variant detection algorithms coupled with a mathematical model we were able to comprehensively evaluate the viral diversity of transmitted/founder viruses in these individuals.

## Results

Using intra-host codon diversity frequencies, coupled with a mathematical model of random virus evolution during acute HIV-1 infection, we were able to unambiguously identify that 30% of subjects exhibited convincing evidence for multiple transmitted/founder viruses in line with prior reports on MSM transmission. Surprisingly, deep sequencing outside of the Env region identified additional low frequency variants possibly reflective of evidence of multiple transmitted/founder viruses in an additional 20% of subjects.

## Conclusion

Our study reveals that, in line with prior reports, approximately 30% of MSM subjects exhibit multiple transmitted/founder viruses, with deep sequencing possibly identifying additional cases of multiple founder viruses which will require further validation. In summary, this data highlights the potential that whole deep sequencing has to uncover additional footprints originating from multiple transmitted/founder viruses. These findings, coupled with the knowledge that dual infections are associated with accelerated disease progression, demonstrate that the higher risk of virus acquisition in MSM could be a greater barrier for vaccine control which may have to contend with multiple transmitted variants.

